# A female patient with Dent disease due to skewed X-chromosome inactivation

**DOI:** 10.1093/ckj/sfae092

**Published:** 2024-04-02

**Authors:** Viola D'Ambrosio, Elizabeth R Wan, Keith Siew, Wesley Hayes, Stephen B Walsh

**Affiliations:** London Tubular Centre, UCL Department of Renal Medicine, University College London, London, UK; Università Cattolica del Sacro Cuore di Roma, Rome, Italy; London Tubular Centre, UCL Department of Renal Medicine, University College London, London, UK; London Tubular Centre, UCL Department of Renal Medicine, University College London, London, UK; London Tubular Centre, UCL Department of Renal Medicine, University College London, London, UK; Great Ormond Street Hospital for Children NHS Foundation Trust, London, UK; London Tubular Centre, UCL Department of Renal Medicine, University College London, London, UK

**Keywords:** Dent disease, Fanconi syndrome, skewed X-chromosome inactivation, tubulopathy, X-linked

## Abstract

X-linked proximal tubulopathies are rare diseases that predominantly affect men. Women are generally carriers and clinical or biochemical manifestations are usually absent or mild. We present the case of a young woman who presented with a full phenotype of Dent disease type 1 due to a de novo mutation in the *CLCN5* gene and a skewed X-chromosome inactivation. Although cases of overt Dent disease type 2 and Lowe syndrome in women have been described in the literature, to our knowledge this is the first case of overt Dent disease type 1.

## INTRODUCTION

We present the case of a 19-year-old girl affected by Dent disease type 1 (MIM 300009) due to a de novo mutation in the *CLCN5* gene with skewed X-inactivation (SXI). Dent disease is an ultrarare X-linked proximal tubulopathy, biochemically manifesting with low molecular weight proteinuria (LMWP), hypercalciuria, glycosuria, aminoaciduria and hyperphosphaturia. Patients can present clinically with nephrolithiasis/nephrocalcinosis, hypophosphataemic rickets and progressive kidney failure. Two types of Dent disease have been identified: type 1 caused by mutations in *CLCN5* and type 2 caused by specific mutations in *OCRL* (other *OCRL* mutations cause Lowe syndrome) that manifests with a Dent-like phenotype. Like other recessive X-linked conditions, Dent disease predominantly affects men, however, heterozygous women can present with a spectrum of clinical manifestations ranging from asymptomatic (carriers) to mild urinary and serum abnormalities (manifesting heterozygotes). Despite being female, our patient presented with overt biochemical and clinical manifestations of Dent disease due to a skewed X-chromosome inactivation (XCI). To our knowledge, this is the first reported case of a female affected by Dent disease type 1 due to XCI.

## CASE REPORT

**Figure 1:**
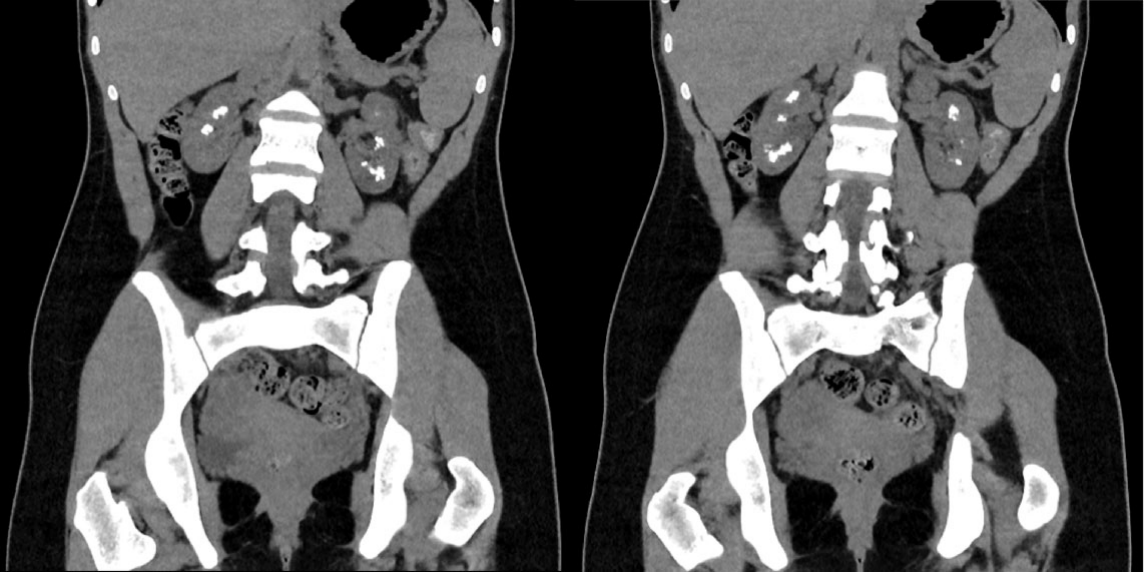
Computed tomography of the kidneys, ureters and bladder showing extensive bilateral medullary nephrocalcinosis.

**Figure 2:**
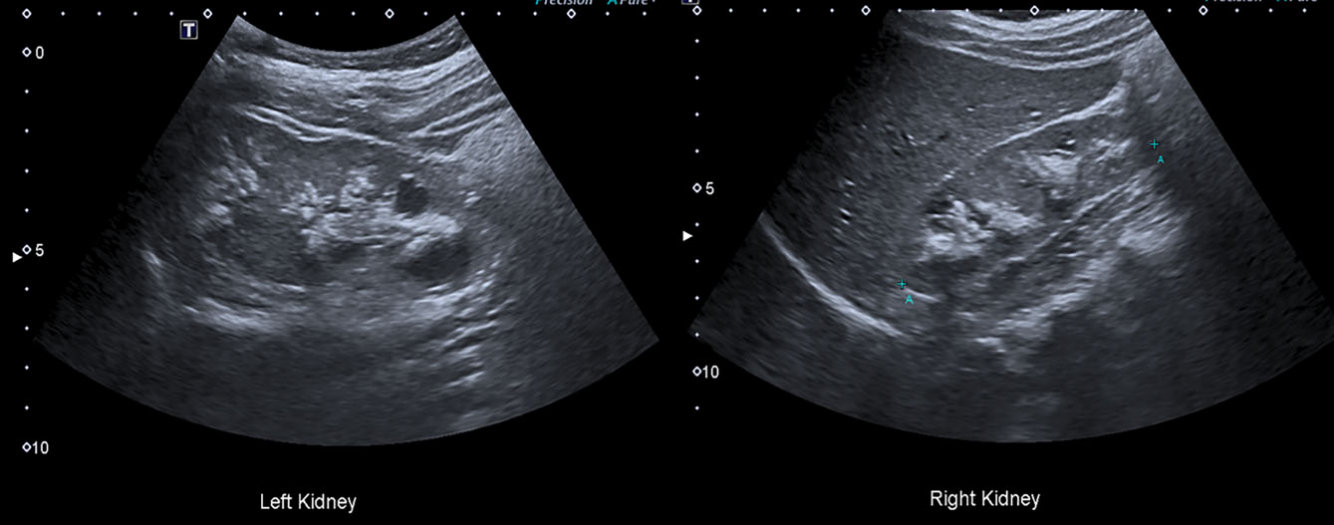
Abdominal ultrasound showing bilateral medullary nephrocalcinosis.

A 19-year-old woman was referred to our tertiary renal tubular service for hyposhosphataemic rickets. Her clinical history started at the age of 2 years when she was diagnosed with rickets initially ascribed to vitamin D deficiency and later to Fanconi syndrome. She had no history of nephrolithiasis, renal colic or urosepsis. Her family history was unremarkable. Her blood pressure was normal and her height was 140 cm. Her serum biochemistry showed impaired kidney function with a serum creatinine of 106 µmol/L, an estimated glomerular filtration rate (eGFR) of 68 ml/min/1.73 m^2^ and hypophosphatemia (serum phosphate 0.73 mmol/L). Her remaining biochemistry was unremarkable. Her urinary metabolic assessment revealed a grossly elevated retinol binding protein (RBP) of 62 900 mg/l (normal range 20-40 mg/l), a urinary RBP:creatinine ratio of 25 160, hypercalciuria with a urinary calcium:creatinine ratio (uCa:Cr) of 1 mmol/mmol (normal range 0.3–0.7 mmol/mmol), hyperphosphaturia with a fractional excretion of phosphate (FePO_4_) of 48% (normal value <20%) and TmP/GFR of 0.62 mmol/l (normal range 0.84–1.23 mmol/l). Computed tomography of the kidneys, ureters and bladder (Fig. 1) and abdominal ultrasound (Fig. 2) showed extensive bilateral medullary nephrocalcinosis.

Her genetic testing, a next-generation sequencing (NGS) panel performed on peripheral lymphocyte DNA, revealed a heterozygous c.2152C>T variant in the *CLCN5* gene (reference sequence: NM_001127899.3). Both parents’ peripheral lymphocyte DNA was tested and was negative for the aforementioned mutation, therefore the variant was classified as de novo. This variant is known to be pathogenic [1–3] and causes substitution of the amino acid arginine with a premature stop codon p.(Arg718*). To explain the clinical manifestation of an X-linked recessive condition in a heterozygous female, X-inactivation studies were carried out. X-inactivation status was studied using polymorphic markers that are differentially methylated on the inactive and active X chromosome. The androgen receptor (AR) was studied by fluorescent polymerase chain reaction in the presence and absence of the methylation-sensitive restriction enzyme HpaII. This revealed a non-random inactivation of the maternally inherited X-chromosome. After 2 years of follow-up the patient remains asymptomatic. Her kidney function has stabilized with no further eGFR decline observed and her plasma phosphate is at the lower limit of normal with oral supplements.

## DISCUSSION

Women with phenotypes of X-linked nephropathies have been described in the literature [4] and can present with various penetration of the disease phenotype because of XCI. XCI is a physiological epigenetic mechanism involving the transcriptional silencing of one of the X chromosomes during the early stages of human embryonic development. XCI remains permanent and it is clonally inherited by daughter cells. XCI is usually random, meaning that females present some cells with the maternal copy of the X chromosome inactivated and some cells with the paternal copy inactivated. This contributes to a phenotypic (but not genotypic) mosaicism in females and recent evidence has also demonstrated that X-inactive specific transcript (Xist)-mediated XCI has an active role in sex-biased autoimmunity [5]. XCI is usually a stochastic mechanism, however, it may be skewed in up to 35% of females. If significantly skewed to expression of an X-chromosome bearing a pathogenic variant, then an X-linked disease may manifest in a female patient. Skewed XCI can be driven by chance or by genes and in case there is one mutated X chromosome, XCI can have a protective or a deleterious effect.

Having only one copy of the chromosome, in X-linked diseases, men typically have more severe clinical manifestations whereas women, due to XCI, can range from being asymptomatic carriers to manifesting heterozygotes. Women affected by Dent disease can therefore be completely asymptomatic or have only biochemical abnormalities. Overt clinical manifestations such as nephrolithiasis, nephrocalcinosis, rickets or chronic kidney disease are extremely rare in manifesting heterozygotes. It would be interesting to know the XCI skewness in heterozygotes manifesting Dent disease. To our knowledge, this is the first case of a woman affected by Dent disease type 1 caused by a de novo mutation in *CLCN5* and skewed XCI and manifesting with an overt phenotype.

## Data Availability

All data underlying the results are available as part of the article.
